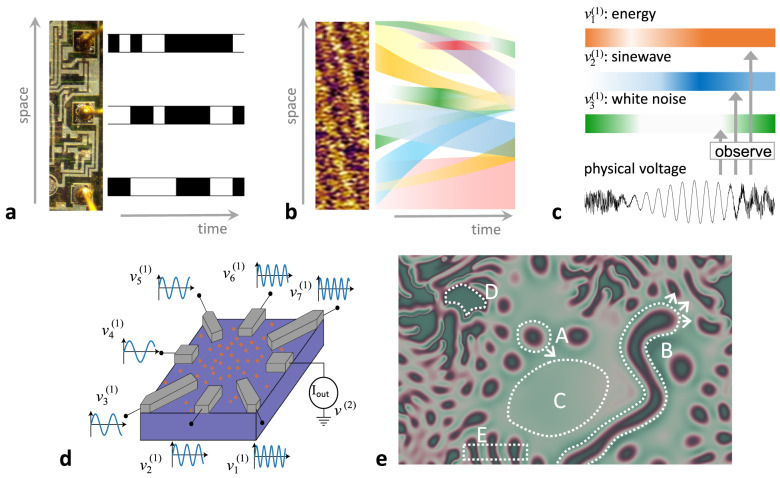# Toward a formal theory for computing machines made out of whatever physics offers

**DOI:** 10.1038/s41467-023-40533-1

**Published:** 2023-08-16

**Authors:** Herbert Jaeger, Beatriz Noheda, Wilfred G. van der Wiel

**Affiliations:** 1 Bernoulli Institute, University of Groningen, 9700 AB Groningen, The Netherlands; 2 Groningen Cognitive Systems and Materials Center (CogniGron), University of Groningen, 9700 AB Groningen, The Netherlands; 3 Zernike Institute for Advanced Materials, University of Groningen, 9700 AB Groningen, The Netherlands; 4 BRAINS Center for Brain-Inspired Nano Systems, University of Twente, 7500 AE Enschede, The Netherlands; 5 MESA+ Institute for Nanotechnology, University of Twente, 7500 AE Enschede, The Netherlands; 6 Institute of Physics, Westfälische Wilhelms-Universität Münster, Münster, Germany

**Keywords:** Computer science, Dynamical systems, Electrical and electronic engineering, Computational methods, Electronic devices

## Abstract

Approaching limitations of digital computing technologies have spurred research in neuromorphic and other unconventional approaches to computing. Here we argue that if we want to engineer unconventional computing systems in a systematic way, we need guidance from a formal theory that is different from the classical symbolic-algorithmic Turing machine theory. We propose a general strategy for developing such a theory, and within that general view, a specific approach that we call fluent computing. In contrast to Turing, who modeled computing processes from a top-down perspective as symbolic reasoning, we adopt the scientific paradigm of physics and model physical computing systems bottom-up by formalizing what can ultimately be measured in a physical computing system. This leads to an understanding of computing as the structuring of processes, while classical models of computing systems describe the processing of structures.

## Abstract (web summary)

Learning from human brains to build powerful computers is attractive, yet extremely challenging due to the lack of a guiding computing theory. Jaeger et al. give a perspective on a bottom-up approach to engineer unconventional computing systems, which is fundamentally different to the classical theory based on Turing machines.

## Introduction

Digital computing technologies are accelerating into a narrowing lane with regard to energy footprint^1^, toxic waste^2^, limits of miniaturization^3^ and vulnerabilities of ever-growing software complexity^4^. These challenges have spurred explorations of alternatives to digital technologies. A fundamental alternative is neuromorphic computing^5^, where the strategy is to use biological brains as role model for energy-efficient parallel algorithms and novel kinds of microchips. We also see a reinvigorated study of other unconventional computing paradigms that search for computational exploits in a wide range of non-digital substrates like analogue electronics^6^, optics^7^, physical reservoir computing systems^8^, DNA reactors^9,10^, or chemical reaction-diffusion processes^11^. These approaches have become branded under names like natural computing, physical computing, in-materio (or in-materia^12^) computing, emergent computation, reservoir computing and many more^13–15^. These lines of study can be seen as belonging together in that they typically are interested in self-organization, energy efficiency, noise robustness, adaptability, statistical dynamics in large ensembles—foci that set these approaches apart from quantum computing, a field that we rather see as a variant of digital computing^16^ and do not further address in this article.

A key objective in these fields is to understand how, given novel sorts of hardware systems made from “intelligent matter”^17^, one can “exploit the physics of its material directly for realizing its operations”^18^. A salient example is the realization of synaptic weights in neuromorphic microchips through memristive devices^19^. In digital simulations of neural networks, updating the numeric effect of a synaptic weight on a neuron activation needs hundreds of transistor switching events. In contrast, when a neural network is realized in a physical memristive crossbar array^20^, one obtains an equivalent functionality through a single voltage pulse applied across the corresponding memristive synapse element.

This principle of direct physical mirroring is not limited to updating single numerical quantities. Complex information-processing operations—like random search in some state space, graph transformations, finding minima in cost landscapes, etc.—can be encoded in terms of complex spatiotemporal physical phenomena in many ways, for instance in oscillations, chaos and other attractor-like phenomena; hysteresis; many sorts of bifurcations and input-induced transits between basins of attraction; spatiotemporal pattern formation; intrinsic noise; phase transitions. In turn, these operations and their spatiotemporal encodings can be used to serve complex cognitive functions like problem solving, attention or memory management. The pertinent literature is so extensive that it defies a systematic survey. Other phenomena and their mathematical reconstructions are more specific, for instance heteroclinic channels and attractor relics^21,22^, self-organized criticality^23–25^ or solitons and waves^26,27^. Fabricating materials with atomic precision is today routinely done worldwide, exploring optical, mechanical, magnetic, spintronic or quantum effects and their combinations, for instance in nanowire networks^24,28^ or skyrmion-based reservoir computing^29^. Physical materials and devices offer virtually limitless resources of physical phenomena for building unconventional computing machines. In contrast to the perfect precision of circuit layouts needed for digital hardware, many usable physical phenomena thrive on disorder and can self-organize into functional behaviour^30,31^. An illustrative example from our own work is presented in Box 1.

It remains, however, unclear how one can make best use of these opportunities. We are lacking a general theory framework that would inform us what specific sorts of computations can be served by different physical phenomena. Under the influence of cybernetics, computational neuroscience, systems biology, machine learning, robotics, artificial life and unconventional computing, our intuitions about computing have broadened far beyond the digital paradigm. Information processing in natural and artificial systems has been alternatively conceptualized and formalized in terms of analogue numerical operations^6,32,33^, probabilistic combinations of bit streams^34,35^, signal processing and control^36–40^, self-organized pattern formation^11,26,41,42^, nonlinear neural dynamics^21,43,44^, stochastic search and optimization^9,45^, evolutionary optimization^46–48^, dynamics on networks^49,50^ or statistical inference^51–53^. From this widened perspective, we investigate the need for, and the chances of, a formal theory for computing systems that directly exploit physical phenomena.

Box 1 Complex spatiotemporal phenomena in thin film materials One of us investigates ferroelectric and ferromagnetic effects in novel computational materials. These materials display an ordered phase, which is responsible for long-term bi-stability, and a disordered phase, in which these properties vanish. Ordering across macroscopic distances gives rise to strongly nonlinear responses to external stimuli. The complexity and sensitivity to external stimuli is maximized at phase transitions^113,114^. Some of the novel materials that we synthesize combine multiple types of interactions (magnetic, electrical, mechanical, chemical) and thus display complex phase diagrams with multiple available phases. Using state-of-the-art thin-film deposition techniques, we can make materials that persist permanently at the edge between two phases, or close enough to a phase transition^115^, such that they can be brought from one phase to the other with low-energy external stimuli^116^.

The image (obtained using conducting atomic force microscopy at room temperature, taken from^117^) shows a network of conducting domain walls in a ferroelectric BiFeO_3_ thin layer. The colour coding in picoampere (pA) indicates the current measured vertically through the film to the ground plate at 3 V probing voltage. The domain walls (yellow) have a much higher conductivity than the regions in between the walls. The areas in between (blue) are insulating ferroelectric domains that can be switched into non-volatile memory states. Structures like this might become used, for example, to encode and dynamically switch the large random bit vectors which are the main representational elements in the hyperdimensional computing paradigm^35^.

The properties of these materials—fine-grained conductivity pathways, local multi-stability with resistive properties that are switchable with minimal energy, many timescales, hierarchical topological structuring—hold many promises for computation in materials. Spatiotemporal processes in a regime close to criticality near a phase transition have been described as an enabling condition for complex information processing dynamics^23,25^.
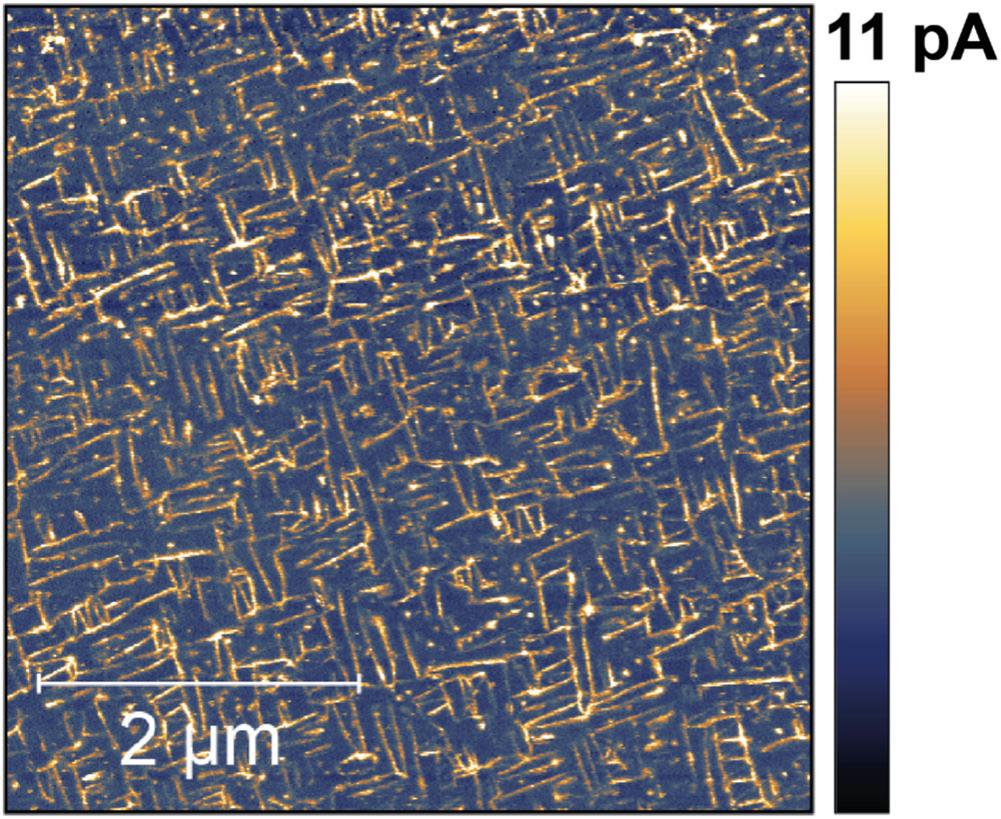

### The algorithmic and cybernetic modes of computing

One might think that we already have a theory of general computing systems, namely what a Turing machine can do. Even philosophers, when they try to come to terms with the essence of computing, invariably orient their argumentation toward Turing computability^54,55^. The mathematical theory of Turing computability constitutes the heart of modern computer science, unleashing a technological revolution on a par with the invention of the wheel. The Turing machine is simulation-universal in the sense that every physical system whose defining equations are known can be simulated on a Turing machine. Given these powers of the Turing paradigm, why should one wish to develop a separate theory for computing with general physical phenomena at all? What could such a theory give us that Turing theory cannot deliver?

We begin with the historical roots of the Turing machine concept. By inventing this formal concept, Turing set the capstone on two millennia of inquiry which started from Aristotle’s syllogistic logic and continued through an uninterrupted lineage of scholars like Leibniz, Boole, Frege, Hilbert and early 20th century logicians. The original question asked by Aristotle—what makes rhetoric argumentation irrefutable?—ultimately condensed in the *Entscheidungsproblem* of formal logic: is there an effective formal method which, when given any mathematical conjecture as input, will automatically construct a proof for the conjecture if it is true, and a counterproof if it is false? While all the pre-Turing work in philosophy, logic and mathematics had finalized the formal definitions of what are conjectures, formal truth, and proofs, it remained for Alan Turing (and Alonso Church^56^) to establish a formal definition of what is an effective method for finding proofs. Turing’s formal model of a general mathematical proof-searching automatism is the Turing machine.

In his ground-breaking article *On computable numbers, with an application to the Entscheidungsproblem*^57^, Turing modelled proof-finding mechanisms as an abstraction of a human mathematician doing formal calculations with paper and pencil. The Turing machine consists of a tape on which a stepwise moving cursor may read and write symbols, with all these actions being determined by a finite-state switching control unit. The tape of a Turing machine models the sheet of paper used by the mathematician (a male in Turing’s writing), the machine’s read/write cursor models his eyes and hands, and the finite-state control unit models his thinking acts. Importantly, Turing speaks of “states of mind” when he refers to the switching states of the control unit—not of physiological brain states. The Turing machine models reasoning processes in the abstract sphere of mathematical logic, not in neural electrochemistry. Students of computer science must do coursework in formal logic, not physiology; and their theory textbooks speak a lot about logical inference steps, but never mention seconds. When one understands the Turing machine as a general model of rational human reasoning, it becomes clear why computers can be simulation-universal: everything that physicists can think about with formal precision can be simulated on digital computers, because they can simulate the physicist’s formal reasoning.

The royal guide for shaping intuitions about the physical basis of computing are biological brains, especially human brains. We will now take a closer look at what aspects of physical neural dynamics are not captured by Turing computability.

We first admit that one specific aspect of brain-based information processing is indeed homologous to Turing computing. Humans can carry out systematic, logically consistent sequences of arguments and planning steps. Let us call this the algorithmic mode of computing (AC). After all, Turing shaped his model of computing after a human mathematician’s reasoning activity. However, our biological brains fall short of the mathematical perfection of Turing machines in many ways. Our ability to construct nested symbolic structures is limited^58^; the concepts that our thinking is made of are not as clean-cut and immutable as mathematical symbols are, but graded, context-varying and incessantly adapting^59,60^; our reasoning often uses non-logical, analogy-based operations^61^. The Turing machine is a bold abstraction of a particular aspect of a human brain’s operation.

Most of the time, however, most parts of our brain are not busy with logical reasoning. In our lives, most of the time we do things like walking from the kitchen table to the refrigerator, without clean logical thinking. Yet, while walking to the refrigerator, the walker’s nervous systems is thoroughly busy with the continual processing of a massive stream of sensor signals, smoothly transforming that input deluge into finely tuned, uninterrupted signals to hundreds of muscles. Let us call this sensorimotor flow of information processing the cybernetic mode of computing (CC). For the largest part of biological history, evolution has been optimizing brains for cybernetic processing—for “prerational intelligence”^62^. Only very late, some animals’ brains acquired the additional ability to detach themselves from the immersive sensorimotor flow and generate logico-symbolic reasoning chains. Several schools of thinking in philosophy, cognitive science, AI and linguistics explain how this ability could develop seamlessly from the cybernetic mode of neural processing, possibly together with the emergence of language^63–68^.

### Aligning and contrasting algorithmic and cybernetic computing

**Fig. 1:**
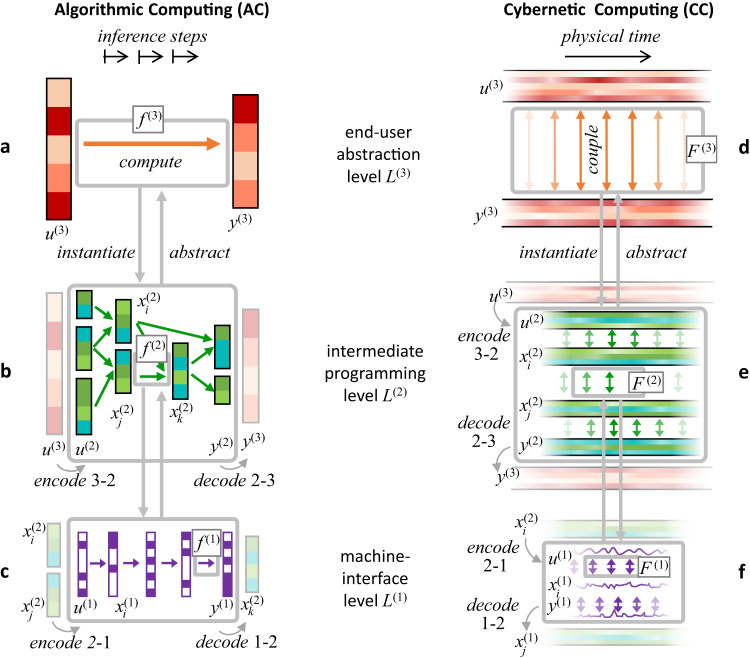
Modeling an algorithmic and a cybernetic computing system on three levels of abstraction. **a**–**c** Digital computing system are typically modelled as algorithmic. The overall functionality of such a system is to transform input data structures *u*^(3)^ into output data structures *y*^(3)^. An algorithmic model thus represents, on the global task level, a mathematical function *f*^(3)^ from inputs to outputs (**a**). To model and implement how this global task function is realized on a digital computing machine, it it stepwise broken down (compiled) into finer-grained models, where data structures (vertical colour bars) and functions (arrows) become hierarchically dissected until at a machine-interface level (**c**), both can be straightforwardly mapped to the digital circuits of the underlying microchip. **d**–**f** Cybernetic computing systems, like brains or analogue processing chips for signal processing and control, transform a continually arriving input signal *u*^(3)^ into an output signal *y*^(3)^ by a continually ongoing nonlinear dynamical coupling *F*^(3)^. Like digital data structures and processes, signals and their couplings can be hierarchically broken down from a global task-level specification (**d**) to machine-interfacing detail (**f**). Symbols *x* represent intermediate data structures (in algorithmic models) and signals (in cybernetic models). Three modeling levels are shown, but there may be more or fewer.

In order to get a clearer view on these two modes of computing, we place two schematic processing architectures side by side (Fig. 1). Each of them is shown with three levels of modeling granularity, from a fine-grained machine interface level *L*^(1)^ via an intermediate level *L*^(2)^ to the most abstract task specification level *L*^(3)^. To make our discussions concrete, we exemplify them with the elementary algorithmic task of multiplying 6 with 5 on a pocket calculator^69^, and the paradigmatic cybernetic task of regulating the speed of a steam engine with a centrifugal governor^70,71^.

We begin with the algorithmic dissection of the multiplication task. Digital computations are modelled (or, equivalently, programmed) by breaking down a user’s task description through a cascade of increasingly finer-granular formalisms down to a low-level machine interface formalism—think of compiling a program written in the task-level machine learning toolbox TensorFlow^72^ down to machine-specific assembler code, passing through a series of programs written in languages of intermediate abstraction like Python or C/C++. In our arithmetics example we condense this to three modeling levels (Fig. 1a–c). On the top level *L*^(3)^, the user specifies task instances by typing the input string *u*^(3)^ = 6 * 5, or 17 * 4 etc. In our graphics the structure of this input is rendered by the cells in the vertical state bars. The user knows that there is a mathematical function *f* ^(3)^ (the multiplication of integers) that is evaluated by the calculator, but the user is not further concerned about how, exactly, this computation is effected. This is taken care of by the designer of the calculator, who must hierarchically disassemble the input 6 * 5 down to a machine-interface level *L*^(1)^.

On an intermediate modeling level *L*^(2)^ one might encode the original input *u*^(3)^ = 6 * 5 in a binary representation (for instance 6 → 1 1 0, 5 → 1 0 1, * → binarymult (leftmost green cell bars *u*^(2)^ in b)). This new input encoding is then processed stepwise with functions *f* ^(2)^ (green arrows), possibly in parallel threads, through sequences of intermediate binary representations ${x}_{i}^{(2)}$, until some binary string representation *y*^(2)^ of the result is obtained, which then can be decoded into the top-level representation *y*^(3)^ = 3 0. One of these functions *f* ^(2)^ could for instance be an addition operation ${x}_{i}^{(2)}+{x}_{j}^{(2)}={x}_{k}^{(2)}$, like binaryadd(1 1 0, 1 0 0 = 1 0 1 0 (inner grey rectangle)).

In a final compilation, this instance of the binaryadd operation might become encoded in a sequence of transformations of 8-bit strings (bytes), whose outcome *y*^(1)^ decodes to ${x}_{k}^{(2)}=$1 0 1 0. This encoding format can be directly mapped to the digital hardware by an experienced engineer. In our schematic diagram we assume that this level specifies binary (Boolean) functions *f* ^(1)^ (violet arrows in c) between 8-bit re-writeable register models (vertical violet bars).

We now turn to our view on hierarchical models of cybernetic information processing systems (Fig. 1d–f). On all modeling levels *L*^(*m*)^, inputs *u*^(*m*)^ and outputs *y*^(*m*)^ are signal streams that are continually received or emitted. These signals can be composed from subsignals—think of a robot’s overall sensory input which might comprise subsignals from cameras, touch sensors, the battery and joint angles. This multi-subsignal makeup is reflected in Fig. 1d–f by the stripes inside the *u*^(3)^, *y*^(3)^, *u*^(2)^, ${x}_{i}^{(2)}$ and *y*^(2)^ bands. The time-varying subsignal values, which we call their activations, are indicated by changing colour intensity. The decomposition into subsignals may be hierarchically continued. In Fig. 1 only the first-level subsignal decomposition is shown.

At the lowest machine-interface level *L*^(1)^ (f), input/output signals *u*^(1)^ and *y*^(1)^ as well as intermediate processing signals ${x}_{i}^{(1)}$ are modelled as evolving in real time $t\in {\mathbb{R}}$. At higher modeling levels, more abstract mathematical models ${\mathfrak{t}}$ of temporal progression may be used, allowing for increasing uncertainty about precise temporal localization (detailed in Section 2.4 in^73^). We reserve the word signal for one-dimensional, real-time signals, and use the word chronicles to refer to possibly multi-modal signals that are formalized with possibly abstract time models ${\mathfrak{t}}$.

In our steam engine governor example, the highest-level input chronicle *u*^(3)^ could be composed of the measured current engine speed *s*^(3)^(*t*) and the desired speed *d*^(3)^(*t*). The output chronicle *y*^(3)^ is the steam valve setting signal *p*^(3)^(*t*). The input and output chronicles are continually connected by a coupling law *F*^(3)^, say by the simple proportional control rule ${\dot{p}}^{(3)}=K\,({d}^{(3)}-{s}^{(3)})$. While in this example the coupling is unidirectional from *u*^(3)^ to *y*^(3)^, in general we admit bidirectional couplings. In the terminology of signals and systems, we admit autoregressive filters for coupling laws. We note that the reafference principle in neuroscience^74^ stipulates that output copy feedback is common in biological neural systems.

An intermediate *L*^(2)^-model would capture the principal structure and dynamics of the governor, using chronicles that monitor speeds, forces, angles etc. of system components like masses, levers, axes, joints etc.

On the lowest level *L*^(1)^, the dynamical couplings between *L*^(2)^-chronicles are concretized to match the specific design of an individual physical governor. For instance, a coupling *F* ^(2)^ between a centrifugal force and a compensating gravitational load force, which would presumably be formalized in level *L*^(2)^ by a differential equation, would be resolved to the metric positioning of joints on lever arms, the weights and sizing and strengths of mechanical parts, etc., leading to fine-grained signals *u*^(1)^, *x*^(1)^, *y*^(1)^ like the current force or velocity components on a specific joint, or—in a high-precision model—measures of temperature or vibration which have an impact on the part’s functioning.

The algorithmic–cybernetic distinction is not a clear-cut either-or division. An intermediate view on computing is adopted in models of analogue computing^6,32,33,75^. They follow the AC paradigm in that a high-level function evaluation task is hierarchically broken down into lower-level flowchart diagrams of sequential function evaluations exactly like in our diagrams Fig. 1a–c. At the same time, they also appear as cybernetic in that their data structures are composed of analogue real numbers, and the functions *f* ^(*m*)^ are evaluated through the continuous-time, coupled evolution of differential operators. Furthermore, the input-output theory of algorithmic computing has been variously extended to sequential processing models, for instance through the concept of interactive computing^76^ and (symbolic) stream computing^77^.

The major similarities and differences between algorithmic and cybernetic models of information processing systems, so far as we have identified them until now, are summarized in Box 2.

Box 2 How algorithmic computing is like, and unlike, cybernetic computing Online, real-time, brain-like, cybernetic transformations from input signal streams to output signal streams is in some ways similar to, and in other ways fundamentally different from algorithmic (digital, symbolic) computing (AC). The similarities justify to classify cybernetic computing (CC) as computing in the first place, while the differences mandate the development of a new formal theory of physical computing. In this box we summarize basic similarities and differences.

**AC and CC are both based on compositional data models**. All data items of AC, from integers to spreadsheet tables, come in the form of hierarchically organized, static symbol structures. The information-carrying, continuously varying signals in CC, which we call chronicles, are likewise hierarchically structured into subchronicles and sub-subchronicles, etc.

**Static and discrete versus temporal and graded data models**. The symbolic data structures in AC, once assembled in a computation, stay immutably the same until they are discretely updated to a new structure by a logical operation. Chronicles and their sub-chronicles have continuously varying signal strengths (called activations) and may fade in and out.

**AC as well as CC are based on hierarchical system models**. Designing and using complex computing systems requires theory-guided methods for breaking down a user’s input-output task specification to a formal model that maps to a machine’s hardware. This is achieved by an abstraction hierarchy of computational models, each of which has its own formal concepts.

**Information processing by stepwise logical inference steps versus continual dynamical coupling**. In an AC computational model, the transformation from input to output data structures occurs in sequences of logical update steps. In contrast, the dynamical coupling between input and output chronicles is inherently parallel and tied to continuous physical time on both ends of the system modeling hierarchy, through the external task source signals and the internal physical dynamics of the machine. In mathematical terms, an AC computation is the evaluation of a function, while a CC computation is the evolution of a dynamical system.
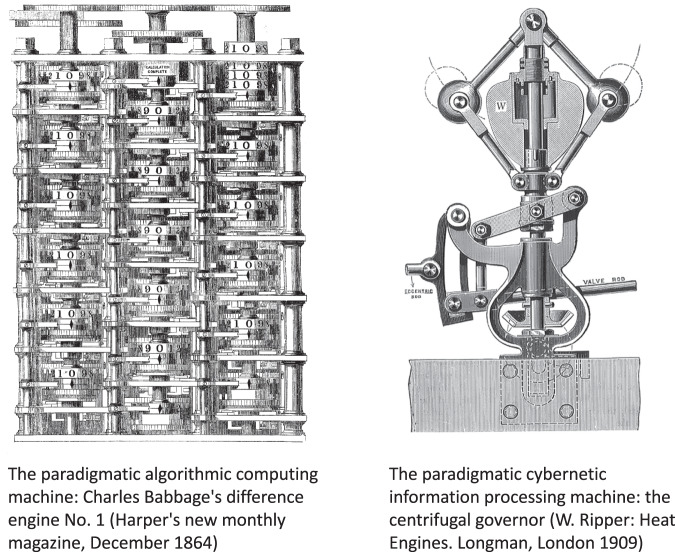

### The core challenge for a theory of physical computing

Working out the general intuitions about modeling computing systems into a concrete theory of physical computing must meet two conflicting demands. First, such a theory must be able to model systems that can exploit a rich diversity of physical phenomena. The ultimate goal is to model systems that are as complex as brains (but might be organized quite differently). Second, such a theory must give transparent guidance to engineers and users of computing systems. The enormity of this double challenge becomes clear when we contemplate how theoretical neuroscience models brains (trying to address the phenomenal diversity demand), and how theoretical computer science models symbolic computation (succeeding in mastering the transparent engineering demand).

We begin with neuroscience modeling. Biological evolution is apt to find and exploit any physiological-anatomical mechanism that adds competitive advantage. Brains appear as “giant ’bags of tricks’ ” which integrate “a huge diversity of specialized and baroque mechanisms”^78^ into a functional whole. Neuroscientists attempt to understand brains on increasingly abstract and integrative modeling levels^79^, from the microscopic biochemistry of synapses to global neural architectures needed for learning navigation maps. Explaining how the phenomena described on some level of abstraction arise from the finer-grained dynamics characterized on the level below often amounts to major scientific innovations. For instance, the Hodgkin-Huxley model of a neuron^80^ abstracts from a modeling layer of electrochemical processes and calls upon mathematical tools from electrical circuit theory; while on the next level of small neural circuits, collective voting phenomena may be explained by abstracting Hodgkin-Huxley neurons to leaky-integrator point neurons and using tools from nonlinear dynamical systems^81^. These ad hoc examples illustrate a general condition in theoretical neuroscience: the price that is paid for trying to understand the phenomenal richness of brains is a diversity of modeling methods.

In contrast, multilevel hierarchical modeling of digital computing processes is done with one single background theory that covers all phenomena within any modeling level as well as the exact translations between adjacent levels. This theory is mathematically rigorous, fits in a single textbook^82^, and lets an end-user of a pocket calculator be assured that their understanding of arithmetic becomes exactly realized by the bit-switching mechanics of their amazing little machine. The price paid is that digital machines can exploit only a single kind of physical phenomenon, namely bistable switching—a constraint that can be seen as the root cause for the problematic energy footprint of digital technologies.

We are certainly not the first ones to take up the challenge of modeling and engineering complex, hierarchically structured cybernetic computing systems. However, we find that none of the proposals that we are aware of fully meets the twofold demand of openness to a broad spectrum of physical mechanisms and unifying engineering transparency across modeling levels. We list four instructive examples. The Neural Engineering Framework^83^, originally developed by Chris Eliasmith and Charles Anderson and used in a sizeable community of cognitive neuroscientists^84–88^, provides mathematical analyses and design rules for modules of spiking neural networks which realize signal processing filters that are specified by ordinary differential equations. This framework does not provide methods for hierarchical model abstraction, and the range of supporting dynamical phenomena is restricted to spiking neurons. Youhui Zhang et al. present a method for engineering brain-inspired computing systems^89^, programming them in a high-level formal design language, which is compiled down through an intermediate formalism to a machine-interface level, which can be mapped to the current most powerful neuromorphic microprocessors. This approach is motivated by practical systems engineering goals and in many ways follows the role model of AC compilation hierarchies. It is however limited to exactly the three specific modeling levels specified in this work, with different principles used for the respective encodings, and at the bottom end exclusively targets digitally programmable spiking neurochips. The Realtime Control System^37^ of James Albus is a design scheme for control architectures of autonomous robotic systems, from the sensor-motor interface level to high-level knowledge-based planning and decision making. Like other models of modular cognitive architectures^90^, it is taken for granted that they are simulated on digital computers. Exploiting general physical phenomena has not been a motivation for their inception. Johan Kwisthout considers Turing machines, which upon presentation of a task input automatically construct a formal model of a spiking neural network that can process this task, and investigates the combined consumption of computational resources for such twin systems^91^. For the neural network model, he allows unconventional resource categories like the number of used spikes. This work makes spiking neural networks accessible to the classical theory of computational complexity, but does not specify how the neural networks spawned by the Turing machine are concretely designed, and the approach is only applicable to a specific formal model of neural networks, not to general physical computing systems.

We are not alone with our impression that we still lack a unifying theory for neuromorphic or unconventional computing. In a 1948 lecture, John von Neumann analysed how computations in brains differ from digital computing. Focusing on stochasticity and error tolerance, he concluded that “we are very far from possessing a theory of automata which deserves that name...”^92^. Similar judgements can be found in the contemporary literature: “The ultimate goal would be a unified domain of all forms of computation, in as far as is possible...”^13^; “As the domain of computer science grows, as one computational model no longer fits all, its true nature is being revealed... new computational theories ... could then help us understand the physical world around us”^93^; “there is still a gap in defining abstractions for using neuromorphic computers more broadly”^94^; “The neuromorphic community ... lacks a focus. [...] We need holistic and concurrent design across the whole stack [...] to ensure as full an integration of bio-inspired principles into hardware as possible”^95^.

In the remainder of this perspective we outline our strategy for developing a formal theory of physical computing. Our aim is to reconcile the two seemingly conflicting modeling demands of capturing general physical systems with their open-ended phenomenology on the one hand, and of enabling practical system engineering on the other. Our strategy is to merge modeling principles that originate in algorithmic and in cybernetic modeling, respectively. From AC we adopt the hierarchically compositional structuring of data structures and processes, which is a crucial enabler for systematic engineering. From CC we take the perspective to view computing systems as continually operating dynamical systems, which enables us to model information processing as the evolution of a physical system. Our key rationale for working out this strategy is to start from physical dynamical phenomena and model computing systems in a hierarchy of increasingly abstracted dynamical systems models, starting from a physics-interfacing modeling level *L*^(1)^. Our name for such formal model hierarchies is fluent computing (FC). This naming is motivated by Isaac Newton’s wording, who called continuously varying quantities “fluentes” in his (Latin) treatise^96^ on calculus.

### A phenomenon is what can be observed

All theories of physics are about phenomena that can be observed (measured, detected, sensed)—at least in principle, and possibly indirectly. In the followship of physics, we want to set up our FC theory such that the values of its state variables can be understood as results of observations (measurements, recordings), and the mathematical objects represented by the variables as observers. We mention in passing that this is the same modeling idea that lies at the basis of probability theory, where random variables are construed as observers of events, too. An observer responds to the incoming signals by creating a response signal. For instance, an old-fashioned voltmeter responds to a voltage input by a motion of the indicator needle. Following the cybernetic view in this regard, we cast observing as a temporal process whose collected observation responses are timeseries objects (chronicles). The time axis of these timeseries may be formalized with modes of progression ${\mathfrak{t}}$ that abstract from physical time $t\in {\mathbb{R}}$. Above we distinguished between input, intermediate, and output variables ${u}^{(m)},{x}_{i}^{(m)},{y}^{(m)}$. For convenience we will henceforth use the generic symbol *v*^(*m*)^ for any of them. We will speak of observers when we mean the objects denoted by model variables *v*^(*m*)^.

Interpreting model variables *v*^(*m*)^ as formal representatives of observers is our key guide for the design of FC model hierarchies, which model a physical computing system in an abstraction sequence that starts with a model at a machine-interface level *L*^(1)^ and ends with a model at a high abstraction level *L*^(*K*)^, which is suitable to express task-specific conditions (as in Fig. 1). A detailed workout of our FC modeling proposal is documented in the long version of this perspective^16^. Here we give a summary of our main ideas.

An observer *v*^(*m*)^ reacts to a specific kind of stimulus with an activation response (think of the readings of a voltmeter or the activation of a visual feature-detecting neuron). This activation ${a}_{{v}^{(m)}}$ may continuously change in time. We admit only positive or zero activation (no negative activations), following the leads of biology (neurons cannot be negatively activated; they can only be inhibited toward zero activation) and the intuition of interpreting activation as signal energy (energy is non-negative). We foresee that relaxed models ${\mathfrak{a}}$ of real-number activations *a* will be needed, with the latter possibly being appropriate only in the physics-interfacing modeling level *L*^(1)^. The general format of an activation at some time at some modeling level would thus be ${{\mathfrak{a}}}_{{v}^{(m)}}({\mathfrak{t}})$.

The specific stimulus part is harder to grasp. We call the specific kind of stimulus to which the observer is responsive, the quality of the observer. However, one cannot exhaustively characterize what a measurement apparatus responds to. Consider a thermometer. While a thermometer is engineered to specifically react to temperature, it will also be sensitive to other physical effects. For instance, it may also react (if only slightly) to ambient pressure, vibration or radiation. In neuroscience, attempts to characterize what exactly a neuron in a brain’s sensory processing pathways responds to remains a conundrum^97^. We do not want to become entangled in this question. Whatever an observer reacts to, we will call the quality of the observer, and we specify this quality by specifying the observer itself. While the activation value of an observer varies in time, its defining quality is unchangeable.

Observers can be composed of sub-observers, and sub-observers can again be compositional objects, etc. For example, a retina can be defined to be composed of its photoreceptor cells, or a safety warning sensor on a fuel tank might be combined from a pressure and a temperature sensor. In our proposed FC terminology, we say that component observers are bound in a composite observer. A plausible binding operator for retina observers would bind the photoreceptor cells through a specification of their spatial arrangement, while the pressure and temperature sensor values might be bound together by multiplication. Many mathematical operations may serve as binding operators. We mention that organizing complex systems models through hierarchically nested subsystem compositions has been a core rationale in complex systems modeling^98^ from the beginnings of that field^99^; that composition hierarchies for data structures and processes are constitutive for AC theory as well as in systems engineering and control^37,100,101^ and the mathematical theory of multi-scale dynamical systems^40,42,102^; and that the so-called binding problem is a core challenge for understanding how cognition arises from neural interactions^100,103–106^. Let us denote the set of observers that are (direct or deeper-nested) components of *v*^(*m*)^ by ${{{{{{{{\mathcal{B}}}}}}}}}_{{v}^{(m)}}^{\downarrow }$, and the set of observers of which *v*^(*m*)^ is a component by ${{{{{{{{\mathcal{B}}}}}}}}}_{{v}^{(m)}}^{\uparrow }$.

Compositional observer-observee hierarchies, where composite observers become tied together by formal binding operators, are the main structuring principle for FC models. They are what we carry over from AC modeling and add to CC modeling. Punchline: AC modeling is based on processing structures, while in FC modeling we structure processes.

Observers can have memory. Their current activation response may depend on the history of what they have observed before. In simple cases, this amounts to some degree of latency needed before the observer’s response settles. In more complex cases, the current activation response can result from an involved long-term integration of earlier signal input—at an extreme end, think of a human who, while reading a novel (observing the text signal), integrates what is being related in the story with their previous life experiences. There are several mathematical ways to capture memory effects. We opt for endowing observers *v*^(*m*)^ with an internal memory state ${{{{{{{{\bf{s}}}}}}}}}_{{v}^{(m)}}({\mathfrak{t}})$, which evolves through a state update operator ${\sigma }_{{v}^{(m)}}$ via update laws of the general format ${{{{{{{{\bf{s}}}}}}}}}_{{v}^{(m)}}(\,{{\mbox{next-}}}\,{\mathfrak{t}})={\sigma }_{{v}^{(m)}}({{{{{{{{\bf{s}}}}}}}}}_{{v}^{(m)}}({\mathfrak{t}}),{({{\mathfrak{a}}}_{{v}^{{\prime} }}({\mathfrak{t}}))}_{{v}^{{\prime} }\in {{{{{{{{\mathcal{B}}}}}}}}}_{{v}^{(m)}}^{\downarrow }})$ In words: the memory state of *v*^(*m*)^ becomes updated by incorporating information from the activations of its component observers. The question how information about previously observed input is encoded and preserved in memory states has been intensely studied, under the headlines of the echo state property and fading memory, in the reservoir computing field^107–112^.

A common theme in complex systems modeling is to capture how the dynamics of subsystems are modulated in a top-down way by superordinate master systems in which the subsystem is a component. This question arises, for instance, in neuroscience where top-down subsystem control serves functions like attention, setting predictive priors, or modulation of motion commands; or in AC programs when arguments are passed down in function calling hierarchies; or in hierarchical AI planning systems where subgoals for subsystems are passed down from higher-up planning systems; or in physics where the interaction of multi-particle systems is modulated by external fields. In our FC proposal we capture top-down control effects by modulating how the activation of a component observer *v*^(*m*)^ is determined by its memory state: ${{\mathfrak{a}}}_{{v}^{(m)}}({\mathfrak{t}})={\alpha }_{{v}^{(m)}}({{{{{{{{\bf{s}}}}}}}}}_{{v}^{(m)}}({\mathfrak{t}}),{({{\mathfrak{a}}}_{{v}^{{\prime}{\prime}}}({\mathfrak{t}}))}_{{v}^{{\prime}{\prime}}\in {{{{{{{{\mathcal{B}}}}}}}}}_{{v}^{(m)}}^{\uparrow }})$, where ${\alpha }_{{v}^{(m)}}$ is the activation function of *v*^(*m*)^.

The dynamics of the memory state ${{{{{{{{\bf{s}}}}}}}}}_{{v}^{(m)}}({\mathfrak{t}})$ and activation ${{\mathfrak{a}}}_{{v}^{(m)}}({\mathfrak{t}})$ are thus co-determined bottom-up by the components of *v*^(*m*)^ and top-down by the master compounds in which *v*^(*m*)^ is a component, respectively, via the state and activation update functions ${\sigma }_{{v}^{(m)}}$ and ${\alpha }_{{v}^{(m)}}$. These interactions are an explicit part of an FC model. They induce implicit interactions between observers, in that two observers may share components or be components in shared compounds. We use the word coupling for these lateral, indirect interactions. Coupling interactions are not explicitly reflected in an FC model. They can, however, have a strong impact on the overall emerging system dynamics. This poses a challenge for the modeler, who must foresee these interactions. This challenge of identifying emerging global organization (or disorganization) phenomena in complex dynamical systems is a notorious problem in all complex systems sciences, and it is also a main problem when it comes to ensure global functionality in complex AC software systems. Two highlight examples: Slotine and Lohmiller^100^ derive formal contractivity conditions for the stability of subsystems such that global system stability (in the sense of recovery from perturbations) is guaranteed; and Hens et al.^40^ analyse how a local perturbation from a globally stable system state spreads in activation waves through a network of coupled subsystems. In this regard, FC modeling faces the same challenges as other complex system modeling approaches.

**Fig. 2:**
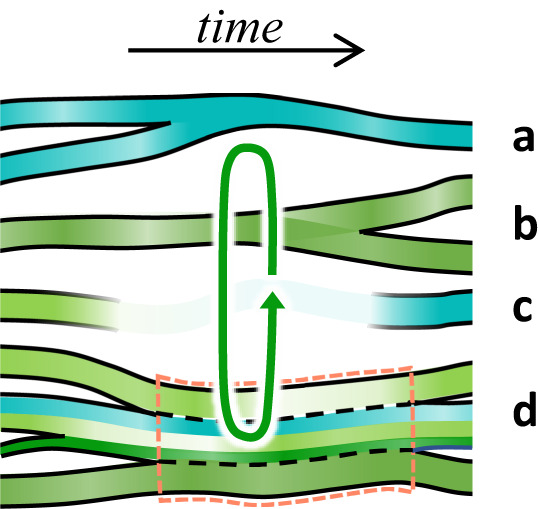
Dynamical re-configuration effects in FC modeling. Observers and their associated chronicles can dynamically bind and unbind in and from compounds during the execution of an FC model, leading to a variety of structural re-organization effects that have obvious analogues in algorithmic computing. From top to bottom: merging (**a**) and splitting (copying, **b**) of observers; termination and creation (**c**); binding and unbinding (**d**). The central segment in **d** (dashed orange outline) shows the temporary presence of a compound observer made from two primitive observers and one compound observer, which in turn is a binding of three primitive ones. The compound observers have activation histories of their own, which are not shown in the graphic. Coupling dynamics is symbolized by the circular arrow.

Binding relations as well as state and activation update functions ${\sigma }_{{v}^{(m)}}$ and ${\alpha }_{{v}^{(m)}}$ can be time-varying, leading to dynamically changing system re-organizations (Fig. 2).

Finally, two models that are adjacent at levels *L*^(*m*−1)^, *L*^(*m*)^ in an abstraction hierarchy of FC models, are related to each other in that some observers *v*^(*m*)^ in level *L*^(*m*)^ can be declared as observing observers *v*^(*m*−1)^ in the model below. This defines a grounding of the level-*L*^(*m*)^ model in the level-*L*^(*m*−1)^ model, and most importantly, a grounding of the machine-interfacing model at level *L*^(1)^ in the physical reality of the underlying physical system. We must refer the reader to our long version^16^ of this perspective for a detailed discussion of consistency conditions for this level-crossing model abstraction and a comparison with model abstractions in traditional computer science and the natural sciences. In Box 3 we point out some facts about observations that are pertinent to building model abstraction hierarchies.

#### Outlook

Research in neuromorphic and other unconventional kinds of computing is thriving, but still lacking a unifying theory grounding. We propose to anchor such a theory in three ideas: viewing information processing as a dynamical system (adopted from the cybernetic paradigm), organizing these dynamics in hierarchical binding compounds (adopted from the algorithmic paradigm), and ground theory abstraction in hierarchies of formal observers (following physics). Surely there are many ways how these ideas can be tied together in mathematical detail. Our fluent computing proposal is a first step in one of the possible directions. We hope that this perspective article (and its long version^16^) gives useful orientation for theory builders who, like ourselves, are searching for the key to unlock the richness of material physics at large for engineering neuromorphic and other unconventional computing systems.

Box 3 Some observations about observing In our project of developing a theory of fluent computing (FC), we interpret the information-carrying model variables as observers (detectors, sensors, measurement apparatuses). Here we point out some aspects of the observer concept that an FC theory should cover.

**a. Localizing bit signals**. An observer—whether a voltmeter or a human—does not observe the world at large but focuses on a specific signal source. One way to identify sources is by spatial localization. The binary bit switching signals in a digital microchip can be picked up at pointlike, non-moving localizations.

**b. Observables can be spatially extended**. In physical substrates (image insert shows the material from Box 1) one can observe objects or phenomena that are spatially extended, geometrically time-varying, and moving—fields, wave fronts, particles and more. An observer must have means to identify and track such objects.

**c. There are unlimited ways to define observables**. The graphic shows response signals from three observers ${v}_{1}^{(1)}$, ${v}_{2}^{(1)}$, ${v}_{3}^{(1)}$ whose common source is the voltage of an electronic contact point. Their activation (indicated by colour intensity) respond to the short-term averaged signal energy, a specific sine frequency response, and the white noise component, respectively.

**d. A complex physical observer**. The drawing (adapted from ref. ^118^) shows a schematic of a dopant network processing unit (DNPU)^31^, an unconventional nanoscale device developed in one of our labs. It consists of a doped silicon well which here is contacted by seven input and one output electrode. DNPUs exhibit strongly nonlinear charge transport behaviour between the electrodes. The drawing shows an experiment where different input voltage signals lead to a nonlinear current response in the output. A neural network was trained to model this highly nonlinear 7-input, 1-output function. Using this model, modular DNPU architectures were mathematically optimized to yield very compact signal processing and pattern recognition systems, including handwritten digit recognition^119^, which were then tested with physical DNPUs.

This neural network model of a DNPU can be formally cast as a level *L*^(2)^ observer of seven signals ${v}_{1}^{(1)}-{v}_{7}^{(1)}$ on the modeling level *L*^(1)^. The activation signal *v*^(2)^ of the trained model output neuron is the response of this observer.

**e. Observing spatiotemporal patterns**. This graphic (background image created with the online tool^120^) shows a snapshot from an evolving chemical reaction-diffusion system. We might consider as observable phenomena, for example, moving solitons (A), moving and growing filaments (B), neutral ground state areas (C), activated areas (D), periodic patterns (E). Arbitrarily more pattern categories can be defined.